# Time to clarify State obligations and accountability on NCDs with human rights instruments

**DOI:** 10.1136/bmjgh-2019-002155

**Published:** 2019-12-09

**Authors:** Kent Buse, Wafa Aftab, Sadika Akhter, Linh Bui Phuong, Haroun Chamli, Minakshi Dahal, Sarah Hawkes, Nousheen Akber Pradhan

**Affiliations:** 1 Political Affairs and Strategy, UNAIDS, Geneva, Switzerland; 2 Department of Community Health Sciences, Aga Khan University, Karachi, Pakistan; 3 ICDDRB, Dhaka, Bangladesh; 4 Center for Population Health Sciences, Hanoi University of Public Health, Hanoi, Vietnam; 5 SURVEN/INNTA, Ministry of Health, Tunis, Tunisia; 6 Center for Research on Environment Health and Population Studies, Kathmandu, Nepal; 7 IGH, University College London, London, UK

**Keywords:** health policies and all other topics

In the Foreword to Benjamin Mason Meier and Lawrence Gostin’s edited volume on human rights and international organisations, Mary Robinson, former United Nations High Commissioner for Human Rights, writes that she first recognised the potential for human rights to contribute to public health in the response to AIDS.^1^

People living with and affected by HIV, and their allies, initially campaigned for rights-based approaches to confront stigma and discrimination. The AIDS movement demanded that their experiences and expertise were at the forefront of the response. ‘Nothing about us without us’ was a rallying cry of activists as they used the Greater Involvement of People living with AIDS principles to champion other rights-based norms including equity, transparency and accountability. Rights-based approaches included investment in legal literacy to empower marginalised communities to address social exclusion, inequalities and injustice and the strategic use of law to pressure governments and corporations to act with urgency. Initially the movement campaigned for individual civil and political rights (eg, privacy, non-discrimination) and with time encompassed economic and social rights (eg, addressing the structural drivers of vulnerability). At its core, the rights-based approach was a political project to race AIDS up agendas as well as enable people to reclaim their humanity and agency.^2^ Importantly, in 2001, the language of human rights was reflected in the United Nations General Assembly *Declaration of Commitment on HIV/AIDS*: the monitoring framework, still implemented, includes rights. UNAIDS (The Joint United Nations Programme on HIV/AIDS) technical support for countries’ periodic reports has been crucial.^3^

Many commentators credit the human rights framework as the bedrock of AIDS progress over the past four decades. Rights-based approaches have been similarly effective in global efforts to curb tobacco consumption and realise reproductive and sexual health. Although demanded by activists, creating an enabling legal environment and addressing the structural constraints to good health, are considered to be the responsibility of the state, with non-communicable diseases (NCDs) now accounting for more than 70% of global deaths each year, and with more than 85% of premature NCD deaths occurring in low-income and middle-income countries.^4^ NCDs have gained greater prominence on many countries’ policy agendas. The first High-Level Meeting of the UN General Assembly on Prevention and Control of NCDs, held in 2011, added significant momentum. In the lead-up to the Meeting, the NCD Alliance published a paper on NCDs and human rights. Among other things, it asserted that ‘The human right to health provides a universal normative framework to design and assess health care and health determinants in relation to NCDs’. Further arguing that ‘The promotion and protection of human rights must be integrated into national NCD policies’.^5^

## Human rights in NCDS policies: the potential yet to be realised

So, to what extent are governments instituting policies to fulfil the state’s responsibility to protect, respect and fulfil human rights in relation to NCD risk factors and the needs of people living with NCDs? As part of a study of dietary NCDs policies in Afghanistan, Bangladesh, Nepal, Pakistan, Tunisia and Vietnam we sought to answer that question. Specifically, we examined relevant national policy frameworks for NCDs and compared these with frameworks for HIV (eg, national strategies and multisectoral plans, etc).

We found that the NCDs policy documents contained relatively few references to rights or rights-based approaches. Afghanistan’s National Strategy for Prevention and Control of NCDs (2015–2020), for example, simply indicates that it is in line with the principles outlined in the National Health and Nutrition Policy 2002–2020 that include the right to health and to nutrition.

Bangladesh’s Multisectoral Action Plan for Prevention and Control of NCDs (2018–2025) does not explicitly refer to rights or rights-based approaches but indicates that ‘All people *should* have access to promotive, preventive and curative, and rehabilitative basic health services’ (emphasis added). Similarly, Nepal’s Multisectoral Action Plan on Prevention and Control of NCDs (2014–2020) suggests that ‘All people particularly the poor and vulnerable *should* have access, without discrimination, to nationally determined sets of needed promotive, preventive, curative, rehabilitative and palliative basic health care services as well as essential, safe, affordable, effective and quality medicines and diagnostics without exposing the users to financial hardships’ (emphasis added). Relevant NCDs policies in Pakistan and Vietnam do not mention rights or rights-based approaches. The same is true of Tunisia, although like in other countries, the right to health writ large is articulated in the country’s constitution.

## Human rights in national HIV strategies: elaborate and extensive

By contrast, the HIV policies revealed a wealth of well-specified and extensive considerations for human rights in terms of more language, reference to specific rights and state obligations. Afghanistan’s National Strategic Plan for HIV (2016–2020) explicitly aims to advance human rights and is ‘grounded on the principles of equity, human rights and social determinants of health’. The Strategy seeks to ‘remove’ stigma and discrimination and create ‘enabling social and legal environments’ that ‘protect the health, education, labor and social rights of PLHIV and support effective prevention among key populations, by ensuring their rights to health’. The Strategy elevates ‘supportive laws and advancement of human rights’ by including it among its four programmatic priorities.

Like Afghanistan, Bangladesh includes rights among the principles of its AIDS response in its 2018–2022 National Strategic Plan, explicitly referencing human rights approaches as essential to reduce vulnerabilities to HIV, while also highlighting ‘various rights such as access to health care, information, confidentiality and privacy, legal rights and gender equity’. The Plan adopts a rights approach ‘to maximize service access by marginalized populations and empower them to be involved in all aspects of the national response. Community mobilization will be ensured to address barriers to service access and build self-esteem among key populations’. References to rights and rights-based approaches are made throughout the Strategic Plan, including in its monitoring framework, and collaboration with the Human Rights Commission is indicated.

Nepal’s HIV Strategic Plan (2016–2021) similarly aims to advance human rights and refers repeatedly to human rights and rights-based approaches (33 times). The Plan was developed with communities of people living with and affected by HIV and one of the working groups focused specifically on human rights. The Plan also refers to the legal needs and protection of people living with and affected by HIV.

Pakistan’s HIV and AIDS Prevention and Treatment Act 2007 is an extensive document outlining the measures protecting the rights of people living with and affected by HIV. Programmatic priorities, targets and activities are outlined in a National Strategy (2017–2021). The document makes numerous references to rights, includes a section on rights-related barriers to services. An ‘implementation strategy’ on stigma and discrimination sets out a range of proposed legal and other measures while an annex is titled ‘Strategic Checklist for Monitoring Integration of Human Rights into HIV & AIDS’. Pakistan’s four provinces also have AIDS strategies; each guided by the principles of human rights and each with a human rights section.

The Tunisian National Strategic Plan for HIV and STIs (2015–2018) embraces human rights as one of its underlying principles and one of four impact result areas. Approximately 50 references are made to rights in the Plan which commits, among other things, to legal reforms to reduce stigma and discrimination, to guarantee the dignity of people living with and affected by HIV as well as their access to services, and to strengthening capacity to understand and address human rights.

Vietnam’s National Strategy on HIV/AIDS prevention and control through 2020 commits to adhere to the principles of ensuring human rights (including the equal rights of people living with HIV) and combating stigma and discrimination. The Strategy commits ‘to regularly organize the dissemination and education about the law on HIV/AIDS prevention and control, attaching importance to regulations on the rights and obligations of HIV-infected people’ and to ‘reviewing, modifying and adding regulations and policies in support of HIV-infected social policy beneficiaries, attaching importance to policies on support for and care of HIV-infected and HIV/AIDS-affected children’. The Strategy further pledges to ensuring no laws reinforce stigma and discrimination.

In summary, in contrast to the expansive recognition and commitment to rights in HIV policies in these countries, human rights language and concepts are largely absent from NCD policies. Cynics may dismiss the inclusion of rights language in HIV policies as political correctness, and critics may object to the comparison of HIV and NCDs (arguing that the latter is less stigmatising). Nonetheless, we contend that policies are important as they clarify state commitments to respond to illness and vulnerability in ways that protect and empower people, while also enabling communities to hold governments to account. Rights-based approaches focus on ensuring that individuals are protected against discrimination, but also commit the State to take positive actions: to provide and promote the conditions where the right to health can be fully realised. It is particularly gratifying to see some of these governments making commitments to engage communities affected by HIV in decision-making, and empowering people living with HIV to assert their interests and to live with dignity. It is regrettable that countries have not yet made similar commitments in relation to NCDs and healthy diets.

## Conclusion

Rights-based national policies for prevention and control of NCDs can strengthen countries’ efforts to address the determinants of NCDs, particularly if they recognise the obligation of states to respect, protect and fulfil people’s right to healthy diets, including its responsibility to govern the commercial and social determinants of healthy diets, using legislative, regulatory and administrative measures at its disposal.^6^ Hence, we welcome the recent call made by 180 experts on WHO and OHCHR (United Nations Office of the High Commissioner for Human Rights) to develop international guidance on human rights, healthy diets and sustainable food systems.^7^ A pubic call is garnering additional signatories to the initiative.^8^

Thirty years after WHO and the (then) United Nations Centre for Human Rights organised the first international consultation to discuss HIV and human rights, we think that it is time for similar consultations on rights, NCDs and healthy diets at country, regional, global levels, so as to enable wider engagement of affected communities and civil society organisations, including consumer protection groups. We would encourage civil society organisations, such as the NCD Alliance, to lead such efforts in collaboration with relevant UN and other organisations.

Undertaking broad consultations on and clarifying state obligations in relation to rights and healthy diets could strengthen the human rights elements of countries’ policy development and practice as well as forge the necessary social movements for rights and healthy diets to ensure the realisation of those rights.

In the interim, we would encourage countries to open their planning processes to ensure the engagement of civil society so that future policies on diets and NCDs can be more rights-oriented, gender-oriented and equity-oriented. We know from the AIDS response, that the struggle for rights is an ongoing one, and discriminatory laws and policies remain in place in too many countries. Nonetheless, the AIDS response has also demonstrated that a rights-based approach can be an effective, indeed essential, tool for progress.
